# Deep learning-based automated quantification system for abdominal aortic calcification: multicenter cohort study for algorithm development and clinical validation

**DOI:** 10.3389/fcvm.2025.1647882

**Published:** 2025-10-21

**Authors:** Zhenhong Shao, Enhui Xin, Lisong Chen, Aie Liu, Chaochao Gu, Aijing Li, Yuning Pan

**Affiliations:** ^1^ Department of Radiology, Cixi People’s Hospital Medical Health Group (Cixi People’s Hospital), Ningbo, Zhejiang, China; ^2^ Department of Radiology, The First Affiliated Hospital of Ningbo University, Ningbo, Zhejiang, China; ^3^ Department of Research and Development, United Imaging Intelligence, Shanghai, China; ^4^ Department of Radiology, Ningbo No. 2 Hospital, Ningbo, Zhejiang, China

**Keywords:** deep learning, abdominal aortic calcification, cardiovascular riskstratification, automated quantification, x-ray image

## Abstract

**Objectives:**

To establish an automated scoring system for abdominal aortic calcification (AAC) to facilitate standardized quantitative imaging analysis in support of clinical decision-making in atherosclerosis management.

**Methods:**

x-ray images of the abdominal aorta were obtained for 2,941 individuals from five medical centers in Zhejiang Province. Calcification severity was graded manually using the Kauppila scoring system, and cases were stratified into three groups based on total calcification burden. The automated assessment framework comprised two sequential components: a lumbar spine segmentation model based on nnUnet and an AAC score regression model based on ResNet. Model development was conducted using 1,737 training cases, with internal validation in 471 cases and external validation in 733 cases from independent centers. A retrospective matched cohort study was conducted in 200 AAC patients from Center B (100 dialysis-dependent and 100 not dialysis-dependent cases), *to investigate associations with major adverse cardiovascular events.*

**Results:**

The developed automated quantification system demonstrated mean absolute errors of 1.686 (internal validation set) and 1.920 (external validation set), with strong correlation to expert ratings (Spearman's *ρ* = 0.923 and 0.888, respectively, both *P* < 0.001). Inter-rater reliability analysis revealed excellent agreement with manual scoring (intraclass correlation coefficients of 0.913 internally and 0.874 externally). Stratification based on calcification severity showed optimal sensitivity for the moderate calcification category (88.6%), with superior specificity for the non/mild (94.2%) and severe (91.5%) categories.

**Conclusion:**

The established automated quantification system for AAC exhibits good assessment efficiency and measurement accuracy, offering a standardized approach to refine cardiovascular risk stratification in clinical practice.

## Introduction

Atherosclerosis (AS), as the core pathological basis of cardiovascular diseases, is a leading cause of disability and all-cause mortality worldwide (1–4). Abdominal aortic calcification (AAC), as a radiological marker of AS (5–7), reflects the dynamic progression of vascular calcification in the atherosclerotic process, and the severity of AAC is significantly positively correlation with the arterial plaque burden. Notably, in patients with chronic kidney disease (CKD), particularly those who require maintenance dialysis, a quantitative AAC score has emerged as a key tool for assessing the risk of a cardiovascular event (8). Research has shown that this AAC score can be used not only for quantitative evaluation of the progression of AS but also as an important independent predictor of adverse cardiovascular events and all-cause mortality (9–11).

For the purpose of clinical AAC scoring, x-ray imaging has become the preferred radiological method for assessing AAC due to its significant advantages related to cost-effectiveness (12, 13). However, the diagnostic efficacy of x-ray–based AAC detection is inhibited by multidimensional technical limitations, including: (1) labor-intensive evaluation: traditional imaging assessment involves a manual, resource-heavy approach, which not only prolongs diagnostic time but also leads to inefficient allocation of medical resources (14); (2) subjectivity and variability: visual assessment methods are susceptible to influence by physician cognitive load and inter-observer variability, resulting in limited diagnostic reproducibility (15); and (3) lack of scalability: in large-scale screening scenarios, conventional methods are incapable of processing high-throughput imaging data due to the absence of an intelligent data processing framework. Therefore, the development of novel imaging-based assessment methods for AAC is critically needed.

Against the backdrop of generational advancements in traditional imaging assessment techniques, artificial intelligence (AI)-powered image analysis is reshaping the paradigm of AAC evaluation. Early breakthroughs were achieved through machine learning models based on dual-energy x-ray absorptiometry (DXA) imaging (16, 17), which enabled automated AAC scoring and advanced cardiovascular disease research. However, clinical AAC assessment currently relies primarily on conventional x-ray images (e.g., lateral lumbar or abdominal radiographs). While anatomical structure overlap is a challenge shared by both lateral lumbar DXA and conventional x-ray imaging, the latter presents greater difficulties for automated analysis due to higher image heterogeneity and the absence of standardized quantitative outputs. Conventional x-rays provide benefits like higher spatial resolution and better soft-tissue contrast (resulting from smaller pixels and higher doses), but their variability remains a significant hurdle.

While convolutional neural networks (CNNs) have demonstrated remarkable success in medical image analysis (18), the studies conducted so far toward an automated AAC scoring system have faced significant limitations. For example, one study reported an end-to-end CNN-based AAC scoring model (15), but its single-center design may limit the model's generalizability across heterogeneous clinical settings.

Unlike existing AAC scoring systems constrained by traditional machine learning (16, 17) or single-center designs (15), the present study aimed to use a multicenter dataset to establish an x-ray–based deep learning framework, which can provide enhanced clinical efficiency and scoring accuracy for physicians.

## Materials and methods

### Data acquisition

We retrieved abdominal aortic radiographs from the picture archiving and communication systems (PACS) of five medical centers in Zhejiang Province and exported images in Digital Imaging and Communications in Medicine (DICOM) format while preserving essential clinical information for analysis.

The following inclusion criteria were applied: age >18 years; availability of lateral abdominal or lumbar radiographs; and image coverage from T12 to S1 vertebrae, with anterior soft tissue thickness exceeding vertebral anteroposterior diameter (ensuring complete visualization of the abdominal aorta from diaphragm to iliac bifurcation).

Cases were excluded according to the following exclusion criteria: structural incompleteness or significant vertebral destruction/compression (L1–L4 anterior vertebral height <2 cm), due to the resultant distortion of anatomical landmarks essential for standardized Kauppila scoring; poor image quality due to artifacts/noise obscuring lumbar vertebral structures; or prominent high-density overlapping shadows in lumbar/aortic regions. The detailed baseline clinical characteristics of the study participants are presented in Table 1.

**Table 1:** Baseline patient characteristics and x-ray image acquisition conditions.

Hospital	A	B	C	D	E	Total	*P* value
Sex							*P* < 0.001, F = 11.97
F, *n* (%)	727	414	79	47	185	1,452	
M, *n* (%)	737	319	105	117	211	1,489	
Age, mean ± SD (years)	70.3 ± 10.9 (23–97)	68.6 ± 11.0 (21–97)	62 ± 13.3 (21–94)	65.7 ± 11.2 (37–89)	62.1 ± 11.2 (32–89)	68 ± 11.6 (21–97)	*P* < 0.001, F = 58.14
CKD, *n*	327	164	184	164	396		
Lumbar lateral	1,137	569	–	–	–		
Company	Philips	Shimadzu	–	–	–	–	
Tube current, mA	400 (400–517)	400 (400–630)	–	–	–	–	
Tube voltage, Kv	85 (85–95)	95 (80–95)	–	–	–	–	
Abdominal lateral	327	164	184	164	396		
Company	Philips	Shimadzu	Philips	Philips	United film	–	
Tube current, mA	320 (320–320)	250 (250–400)	320 (320–320)	320 (320–320)	400 (400–508)	–	
Tube voltage, Kv	85 (85–85)	80 (80–100)	85 (85–85)	85 (85–85)	85 (85–85)	–	
AAC score (manual)							
No/Mild	3 (0,4)	2 (0,4)	1 (0,4)	2 (0,4)	2 (0,4)	2 (0,4)	*P* *<* 0.001, F = 12.03
Moderate	9 (5,15)	8 (5,15)	9 (5,15)	7 (5,15)	10 (5,15)	9 (5,15)	*P* *<* 0.001, F = 6.23
Severe	18 (16,24)	18 (16,24)	16 (16,19)	17 (16,23)	19 (16,24)	18 (16,24)	*P* = 0.026, F = 2.86

This study was approved by the Medical Ethics Committee of Ningbo University Affiliated First Hospital (Approval No.: 2025-040A) and adhered to the principles outlined in the Declaration of Helsinki. All clinical data were anonymized per institutional protocols, in compliance with China's Ethical Review Measures for Life Sciences and Medical Research Involving Human Subjects. The need for an informed consent was exempted due to the retrospective nature of the study.

### Dataset partitioning

To reduce computational costs while maintaining accuracy, we randomly selected 142 images from Center A (November 2021 to December 2021) and Center C (November 2023 to November 2023) to establish a small-scale dataset for lumbar segmentation model development. This subset was used for preliminary region-of-interest (ROI) localization, with a 132:10 training:test split ratio. A radiologist manually annotated L1–L5 vertebral bodies using ITK-SNAP software.

To develop the AAC scoring regression model, the dataset was systematically partitioned by lumbar segmentation model. The training set comprised data from: Center A (January 2019 to July 2023), Center C (January 2022 to November 2023), Center D (January to December 2023), and Center E (January 2021 to December 2023). The internal validation set included cases from: Center A (August 2023 to February 2024) and Center E (January to March 2024). We maintained an approximate 4:1 ratio between training and internal validation sets. Additionally, cases from Center B (January 2021 to January 2024) served as the external validation set for rigorous evaluation of the model's generalizability.

### Manual AAC scoring

For manual scoring of AAC, this study employed the internationally recognized Kauppila semi-quantitative scoring method (19, 20), the gold standard for AAC assessment. The standardized evaluation protocol assessed four aortic segment pairs (anterior/posterior walls) corresponding to the L1–L4 vertebral levels, with segment boundaries defined at midpoints of adjacent intervertebral spaces. The grading criteria were as follows: no detectable calcification: 0 points; calcification length <1/3 of aortic segment: 1 point; calcification length ≥1/3 but ≤2/3 of segment: 2 points; and calcification length >2/3 of segment: 3 points. Based on the total score, cases were classified as: no or mild AAC (0–4 points), moderate AAC (5–15 points), or severe AAC (16–24 points) (21).

**Figure 1:**
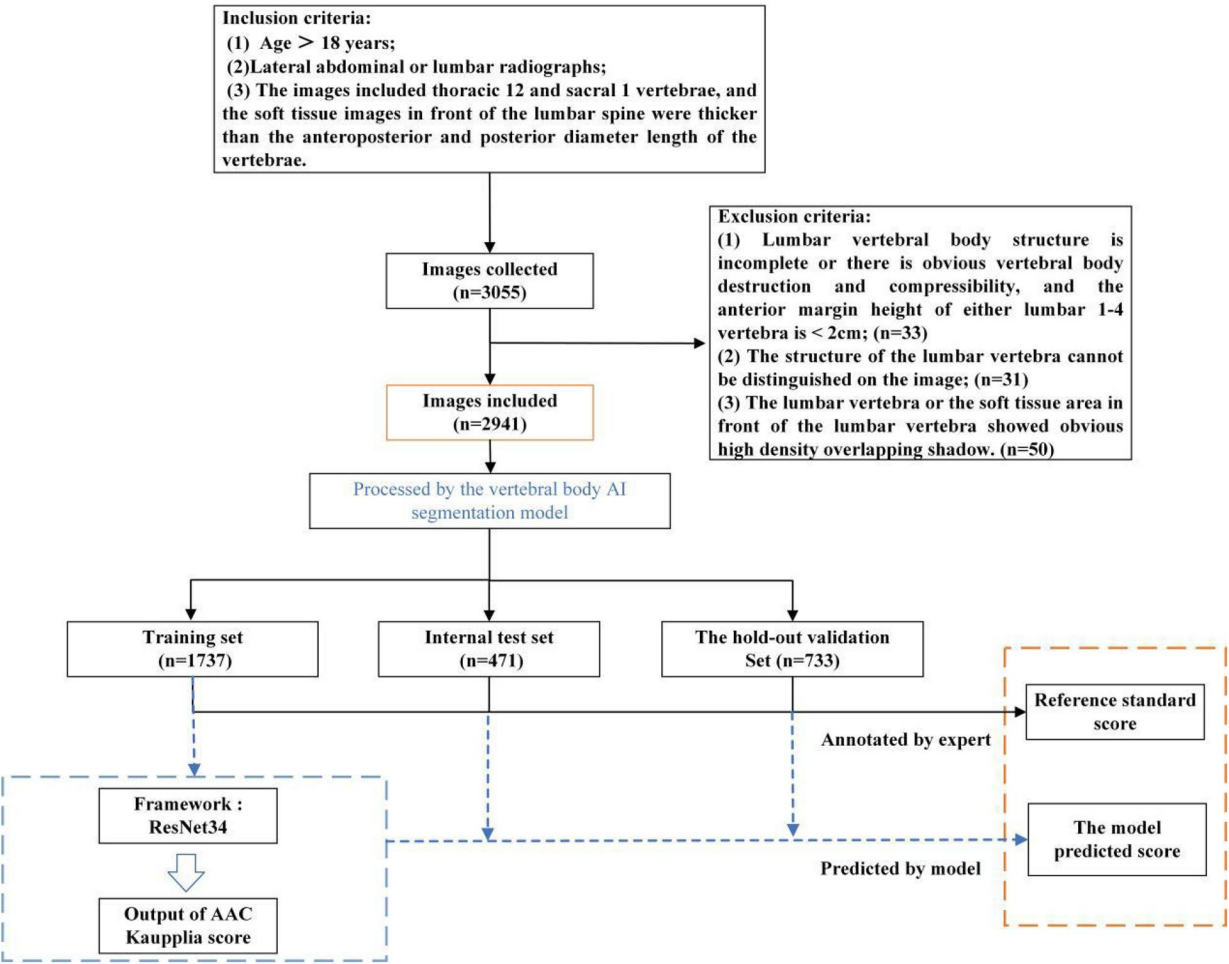
Schematic diagram of the model design and laboratory workflow design processes.

To ensure scoring accuracy, all raters were rigorously trained by applying the Kauppila scoring system to 451 training cases before formal evaluation for the present study. AAC scoring was performed through a standardized, double-blind protocol involving three stages: (1) initial independent scoring by two junior radiologists with <5 years of experience; (2) adjucation by a senior radiologist (≥5 years' experience) for cases with discrepant AAC24 total scores between junior radiologists were adjudicated (Through independent review, the senior radiologist assigned Kauppila sub-scores (0–3) to anterior/posterior walls of L1–L4 vertebrae. The definitive AAC24 reference standard was derived from this assessment.); and (3) quality control verification by another senior radiologist who independently scored cases in the internal/external validation sets and confirmed proper ROI localization in images, which had been processed by the lumbar spine segmentation and localization model. Figure 1 outlines the model design and laboratory workflow design.

### Clinical analysis

This study utilized an artificial intelligence-assisted calcification scoring system to screen abdominal aortic x-ray images from Center B's database (January 2023 to January 2024), identifying cases with AAC positivity (AAC score ≥1). Using a stratified random sampling method based on patients' dialysis history, the study established Observation Cohort 1 comprising 100 patients who received maintenance dialysis. For the control group (Observation Cohort 2), 100 patients not receiving dialysis treatment were chosen based on criteria including a glomerular filtration rate (GFR) >80 ml/min and no history of dialysis. Clinical data for both groups were retrospectively collected through the review of electronic medical records and telephone follow-ups, covering the period from January 2020 to December 2024. The primary focus was comparing the cumulative incidence of major adverse cardiovascular events (MACEs) between the two cohorts during the 5-year follow-up period. The MACE composite endpoint included four clinical outcomes: hemorrhagic stroke, ischemic stroke, myocardial infarction, and heart failure.

### Construction of the AAC automated scoring system

**Figure 2:**
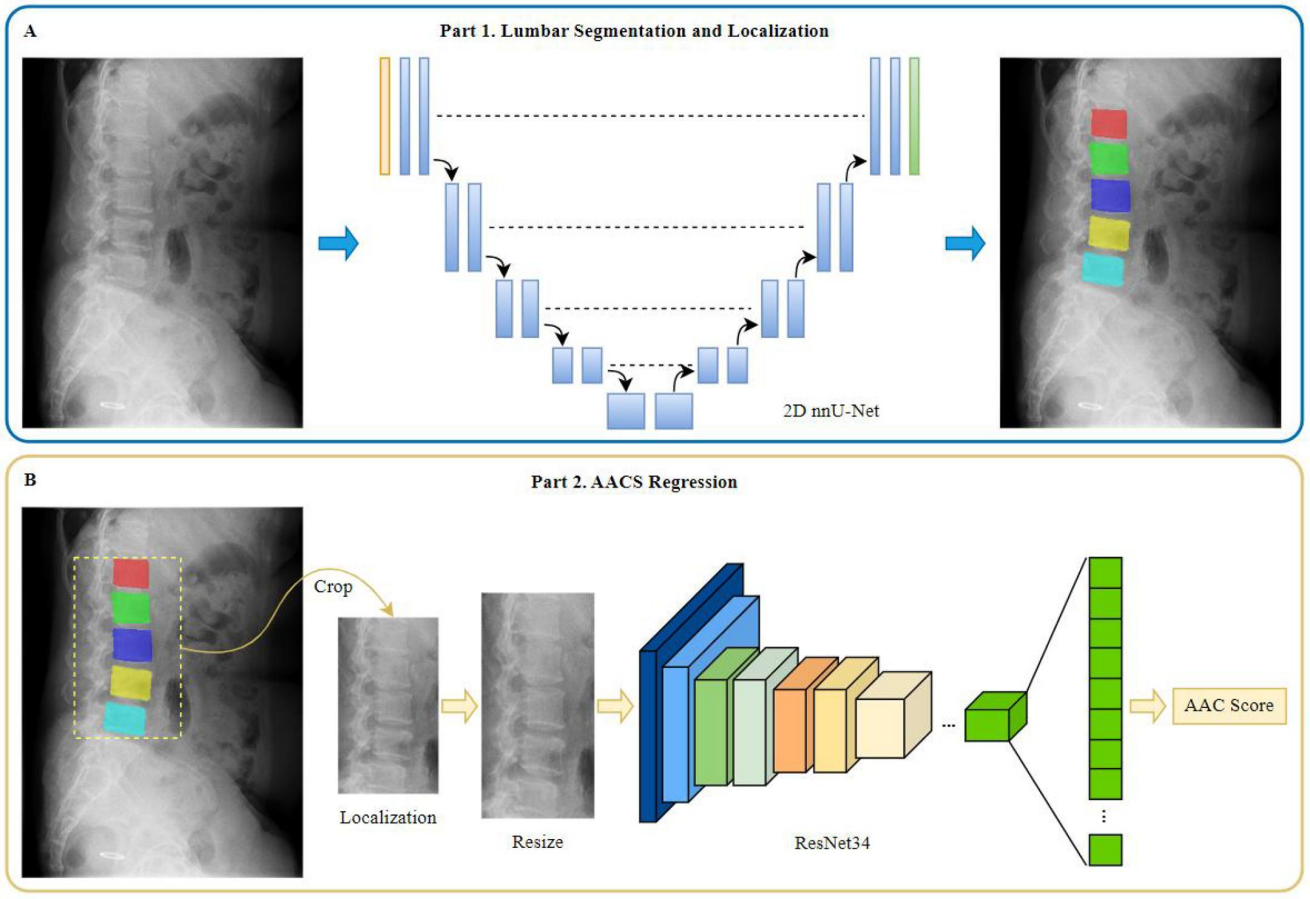
Diagram of model construction for different tasks. **(A)** Part 1 of the model for lumbar segmentation and localization. **(B)** Part 2 of the model for AACS regression.

The AAC automated scoring system primarily consists of two components: a lumbar spine segmentation and localization model and an AAC scoring regression model, as illustrated in Figure 2. The AAC score was treated as a continuous target variable for regression. This approach is justified as the score is derived from a continuous underlying physical quantity (calcified area), and each unit increment represents a comparable change in the extent of calcification.

### Lumbar spine segmentation and localization model

The lumbar spine segmentation model was constructed using the 2D nnU-Net (22) framework, a state-of-the-art tool that automatically configures optimal preprocessing, network architecture, training, and postprocessing for a given dataset. This “no-new-Net” philosophy was selected to eliminate subjective manual tuning and ensure a robust, reproducible baseline, which is critical for providing high-quality masks for subsequent analysis.

The automatically configured architecture featured a 9-level encoder-decoder structure with feature channels of [32, 64, 128, 256, 512, 512, 512, 512, 512] at each stage. The encoder employed consecutive convolutional layers with 3 × 3 kernels and instance normalization, progressively reducing spatial dimensions while increasing feature channels to capture hierarchical features at different scales. The decoder then upsampled the feature maps, recovering the original spatial dimensions of the input image. Skip connections were incorporated to fuse low-level spatial features from the encoder with high-level semantic features from the decoder.

During training, the model was optimized using a combined dice and cross entropy loss based on five-fold cross validation. The network was trained on 1,536 × 1,024 pixel patches with a batch size of 2, employing Z-score normalization for intensity standardization. Complete configurations are provided in the Supplementary plans.json File.

### AAC scoring regression model

Prior to constructing the AAC scoring regression model, a series of image preprocessing steps were performed as follows: (1) segmentation mask generation: using the lumbar spine segmentation and localization model, segmentation masks for vertebrae L1–L5 were obtained; and (2) extraction: based on these masks, normalized original images were cropped to generate image patches containing the L1–L5 vertebral regions. The vertical coverage extended from the inferior border of T12 to the inferior border of L5. The horizontal coverage included the L1–L5 vertebrae as well as soft tissue regions extending 3/4 of the average anteroposterior vertebral length anteriorly. The processed image data were then used as input for the subsequent AAC scoring regression model.

Images were uniformly resized to 500 × 1,000 pixels. To generate a larger, more complex, and diverse dataset for enhancement of model accuracy and generalizability, images were augmented using several different methods, including random brightness adjustment (±10% adjustment), random contrast adjustment (±10% adjustment), random addition of Gaussian noise (σ∈[0,10]), random shift operation (±5% of image size), random scaling (±5%), and random angle rotation (±30 degrees). Augmentation was performed only in the training set before each epoch.

The AAC scoring regression model was constructed based on the framework of ResNet34, a publicly available deep residual network (23) comprising 34 layers. This choice was based on ResNet's established efficacy as a deep feature extractor for two-dimensional images, particularly its ability to mitigate vanishing gradients through residual connections, which enables stable training of deep networks. To validate the architectural choices, a comparative ablation study was conducted against a VGG16_bn-based model under identical training conditions (detailed description provided in Supplementary Materials).

Transfer learning was implementded by fine-tuning the ImageNet-pretrained ResNet34 model weights. While the original ResNet34 was designed for classification tasks with a final fully-connected layer outputting 1,000 class probabilities, we modified the architecture for our regression task by replacing the final classifier with a custom sequential module. This module consisted of batch normalization, a dropout layer (*P* = 0.5), and a linear layer configured to produce a single continuous output value. To enhance feature representation, we integrated Spatial and Channel Squeeze & Excitation (SCse) attention modules (24) after each residual block, enabling the model to focus on informative spatial regions and channel features.

The model was optimized using stochastic gradient descent with a momentum of 0.9 and weight decay of 0.0005, with an initial learning rate of 0.01. Training was conducted with a batch size of 32 for 500 epochs using mean squared error (MSE) as the loss function, with early stopping based on validation performance. Validation loss was monitored every 10 epochs, and training was effectively terminated at epoch 400 when validation loss plateaued, indicating no further improvement (Supplementary Figure S2). We employed an enhanced learning rate scheduling strategy combining linear warmup and cosine annealing. During the initial 200 iterations, the learning rate was linearly increased from 0.0033 (one-third of the base learning rate 0.01) to the full base value of 0.01. This warmup phase improved training stability by preventing early gradient explosion. Subsequently, the learning rate followed a cosine decay function to a minimum value of 0.0001 over the remaining training epochs, facilitating convergence to better optima (25).

### Experimental evaluation metrics

All statistical analyses were conducted using SPSS software (version 27.0) and Python. The normality of continuous variables was assessed using the Shapiro–Wilk test and Q-Q plots. Normally distributed data are presented as mean ± standard deviation (mean ± SD), and non-normally distributed data are reported as median (range or interquartile range [IQR]). Categorical variables are expressed as frequency count (percentage, *n* [%]).

The AAC total score was treated as a continuous variable. Depending on the data distribution (assessed by skewness and kurtosis tests) of both model-predicted scores and manual standard scores, the Pearson correlation coefficient was calculated to evaluate linear relationships when normality assumptions were met. Otherwise, Spearman's rank correlation coefficient was determined to assess monotonic associations. Model fit was quantified using the *R*^2^ coefficient. Prediction accuracy was measured by MSE and mean absolute error (MAE), with 95% confidence intervals (CIs) derived via bootstrapping (1,000 iterations). This involved repeatedly resampling the training set with replacement, retraining the model, predicting on the original test set, and calculating error metrics per iteration. The 95% CIs were defined as the 2.5th and 97.5th percentiles of the resulting MSE and MAE distributions. Agreement between manual standard scores and model predictions was evaluated by calculating intraclass correlation coefficients (ICCs, two-way random-effects model ICC [2,1]) and weighted Kappa coefficients, with statistical significance set at *P* < 0.05.

Model performance was comprehensively evaluated through: (1) confusion matrices and calculation of overall accuracy rates, and (2) subgroup-specific metrics including accuracy, sensitivity, specificity, negative predictive value (NPV), and positive predictive value (PPV). Sensitivity analyses for cardiovascular outcomes included age/sex stratification and E-value calculation to assess unmeasured confounding effects.

## Results

### Baseline characteristics of study participants

The study population included a total of 1,464 cases from Center A (January 2019 to February 2024), 733 cases from Center B (January 2021 to January 2024), 184 cases from Center C (January 2022 to November 2023), 164 cases from Center D (January to December 2023), and 396 cases from Center E (January 2021 to March 2024).

For this study, a total of 1,737 cases were assigned to the training cohort, 471 to the internal validation cohort, and 733 to the external validation cohort. All imaging cohorts retained the full AAC spectrum (0–24). The detailed data distribution is shown in Table 2.

**Table 2:** Dataset partitioning for development and testing of AAC scoring regression model.

Dataset	Total	Abdominal lateral	Lumbar lateral	No/mild AAC (score 0–4)	Moderate AAC (score 5–15)	Severe AAC (score 16–24)
Training	1,737	795	942	387	1,124	226
Internal validation	471	285	186	116	292	63
External validation	733	164	569	246	406	81

### Segmentation and locoalization performance

For the lumbar spine segmentation model, the performance in segmenting vertebrae L1–L5 in the test set was excellent, with Dice scores of 0.92, 0.93, 0.95, 0.94, and 0.94, respectively. The segmentation model was subsequently applied to all imaging data to extract image patches for input into the AAC scoring regression model. Through manual review, the segmentation outputs met predefined ROI coverage criteria in 92.6% of cases (1,115/1,204) across both the internal and external validation sets.

### AAC scoring regression performance

**Figure 3:**
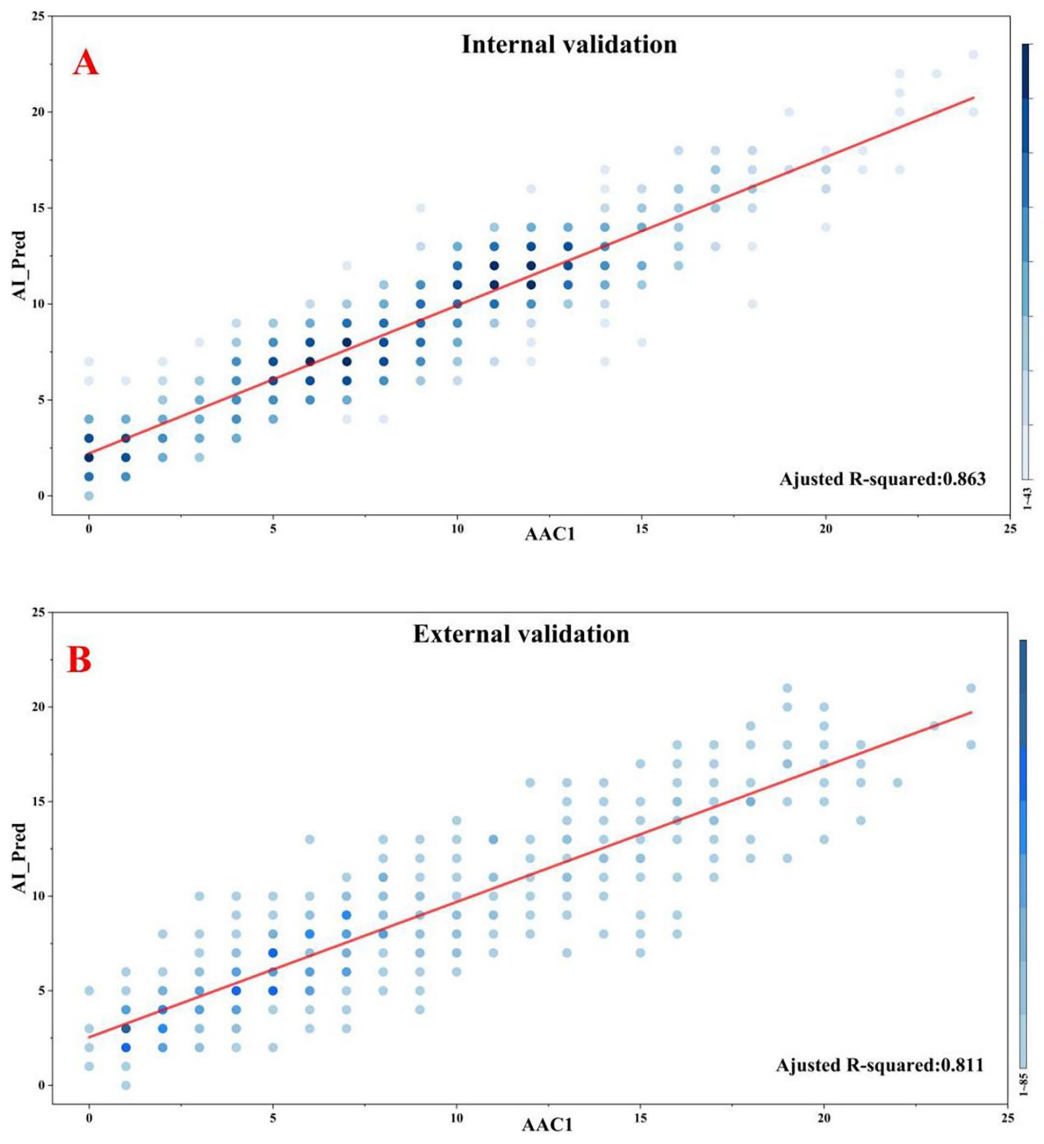
Correlations between model predictions and manual standard scoring. **(A)** Internal validation. **(B)** External validation.

The AAC scoring regression model demonstrated excellent performance on the internal validation set with a MAE of 1.686, MSE of 4.730, and Spearman correlation coefficient of 0.923 (*P* < 0.001). Similar robust performance was observed in the external validation set (MAE = 1.920; MSE = 5.835; Spearman's *ρ* = 0.888, *P* < 0.001). Furthermore, the proportions of predictions deemed clinically acceptable (absolute error <4) were 90.0% and 87.6% in the internal and external validatiaon sets, respectively. Although marginally higher errors were noted in the external validation set, the model maintained clinically acceptable accuracy and stability overall. The complete results for all evaluation metrics are presented in Table 3, with visualizations provided in Figure 3.

**Table 3:** Model performance metrics for accuracy and correlation.

Dataset	Mean absolute error (MAE)	Mean squared error (MSE)	Spearman's *ρ*	*R*^2^ coefficient
Internal validation set	1.686 (1.571, 1.811)	4.730 (4.100, 5.393)	0.923 (0.907, 0.935) *P* < 0.001	0.863 (0.835, 0.887) *P* < 0.001
External validation set	1.920 (1.809, 2.027)	5.835 (5.235, 6.486)	0.888 (0.886, 0.913) *P* < 0.001	0.811 (0.781, 0.835) *P* < 0.001

Comparatively, the results of the ablation study based on an alternative VGG16_bn architecture were calculated. Under identical training conditions, the ResNet34-based model achieved lower MAE and MSE for both the internal and external validation sets (Supplementary Table S1). Furthermore, a higher proportion of predictions from the ResNet-34 model was clinically acceptable (absolute error <4). These results confirm the superiority of the chosen architecture for this task.

To further validate our findings, we conducted a comprehensive agreement analysis between manual standard scores (denoted as AAC1) and model-predicted scores (AI_Pred), with particular focus on ICCs and weighted Kappa statistics. Additionally, we incorporated a second independent manual rating (AAC2) for comparative analysis, to enable robust evaluation of discrepancies between the algorithmic and human assessments.

In the internal validation set, the model-predicted scores (AI_Pred) demonstrated strong agreement with manual standard scores (AAC1), as evidenced by an ICC of 0.913 and a weighted Kappa value of 0.716. For comparison, the inter-rater reliability between two human evaluators (AAC1 vs. AAC2) showed even higher concordance, with an ICC of 0.985 and weighted Kappa of 0.904.

In the external validation set, the agreement between manual standard scores (AAC1) and model predictions (AI_Pred) remained robust, with an ICC of 0.874 and weighted Kappa value of 0.644. By comparison, the inter-observer agreement between human raters (AAC1 vs. AAC2) in the external validation set showed superior consistency (ICC = 0.981, weighted Kappa = 0.887). The detailed results of this analysis are presented in Table 4.

**Table 4:** Comparison of ICC and Kappa values between manual standard scores (AAC1) vs. alternative manual ratings (AAC2) and model predictions (AI_Pred).

Comparison	Dataset	ICC (95% CI)	Weighted Kappa (95% CI)
AAC1 vs. AAC2	Internal validation set	0.985 (0.981, 0.987)*	0.904 (0.891, 0.917)*
	External validation set	0.981 (0.978, 0.983)*	0.887 (0.876, 0.898)*
AAC1 vs. AI_Pred	Internal validation set	0.913 (0.897, 0.927)*	0.716 (0.691, 0.741)*
	External validation set	0.874 (0.854, 0.892)*	0.644 (0.620, 0.667)*

* All *P* < 0.001.

### Model performance according to AAC severity

**Figure 4:**
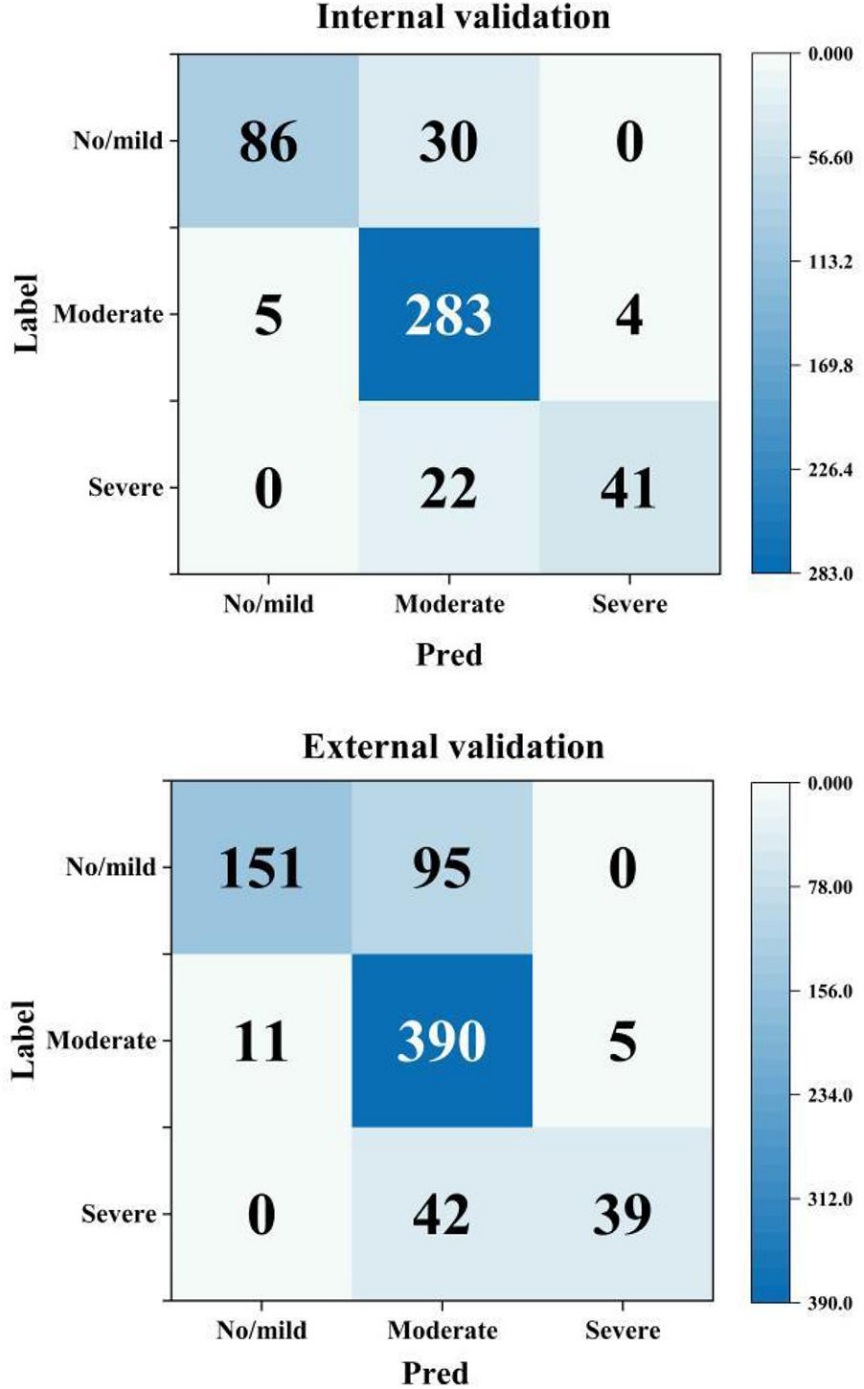
Confusion matrices by AAC severity category (Pred: model prediction, label: manual standard score).

Subgroup analysis revealed significant performance disparities across different AAC severity categories (detailed in Table 5 and Figure 4). Most notably, for both the No/mild (0–4) and Severe (16–24) AAC groups, the model exhibited markedly lower sensitivity compared to that for the Moderate (5–15) category. In the internal validation set, the model had sensitivities of 74.1% for No/mild AAC and 65.1% for Severe AAC vs. 96.9% for Moderate AAC. Similarly, in the external validation set, these sensitivity values were 61.4% for No/mild AAC and 48.1% for Severe AAC vs. 96.1% for Moderate AAC. This consistent pattern (*P* < 0.001 for all inter-group comparisons) suggests potential detection challenges for extreme calcification conditions that warrant clinical attention.

**Table 5:** Model performance metrics across categories of AAC severity.

Internal validation set (*n* = 471)	No/mild AAC (*n* = 116)	Moderate AAC (*n* = 292)	Severe AAC (*n* = 63)
Accuracy (%)	92.6 (86.4–96.1)	87.0 (82.7–90.4)	94.5 (85.9–98.0)
Sensitivity (%)	74.1 (65.4–81.2)	96.9 (94.2–98.4)	65.1 (52.8–75.7)
Specificity (%)	98.6 (94.4–99.7)	70.9 (65.4–75.8)	99.0 (92.5–99.9)
NPV (%)	92.1 (85.7–95.8)	93.4 (90.0–95.7)	94.8 (86.3–98.1)
PPV (%)	94.5 (88.7–97.4)	84.5 (79.9–88.2)	91.1 (81.5–96.0)
External validation set (*n* = 733)	No/mild AAC (*n* = 246)	Moderate AAC (*n* = 406)	Severe AAC (*n* = 81)
Accuracy (%)	85.5 (80.6–89.4)	79.1 (74.9–82.8)	93.6 (86.1–97.2)
Sensitivity (%)	61.4 (55.2–67.3)	96.1 (93.7–97.6)	48.1 (37.6–58.8)
Specificity (%)	97.7 (95.0–99.0)	58.1 (53.2–62.8)	99.2 (94.0–99.9)
NPV (%)	83.4 (78.2–87.5)	92.2 (89.2–94.4)	93.9 (86.4–97.4)
PPV (%)	93.2 (89.3–95.7)	74.0 (69.5–78.0)	88.6 (79.9–93.8)

Regarding specificity, the model showed significantly higher performance for both the No/mild and Severe AAC categories compared with the Moderate category. In the internal validation set, the model's specificity reached 98.6% for No/mild AAC and 99.0% for Severe AAC, whereas it was only 70.9% for Moderate AAC. Similarly, in the external validation set, the model's specificity was 97.7% for No/mild AAC and 99.2% for Severe AAC, in stark contrast with a value of only 58.1% for Moderate AAC (all inter-group comparisons *P* < 0.001). This inverse pattern related to the sensitivity results suggests a potential trade-off effect in model performance across the categories of AAC severity.

### Visual interpretation analysis

**Figure 5:**
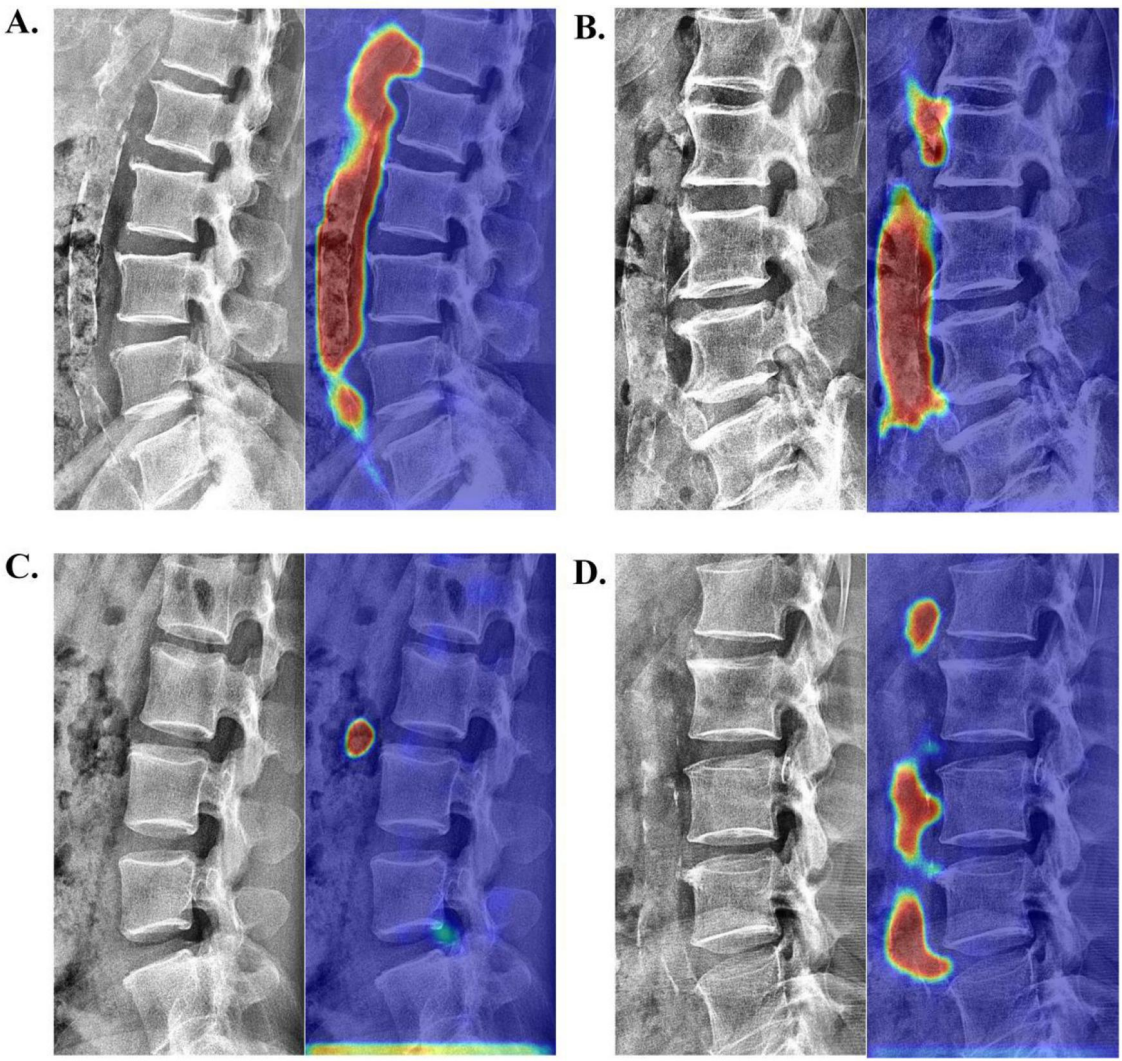
Visualization of neural network activation maps from the developed automated AAC scoring system for four randomly selected representative cases with distinct cardiovascular risk profiles involving: **(A)** chronic kidney disease (manual AAC score: 23; model-predicted score: 22), **(B)** hypertension (manual AAC score: 19; model-predicted score: 17), **(C)** hyperlipidemia (manual AAC score: 1; model-predicted score: 1), and **(D)** coronary artery disease (manual AAC score: 10; model-predicted score: 11).

To qualitatively verify the model's accuracy in localizing calcifications, we employed Grad-CAM (26) for visual explanatory insights. The results demonstrated strong agreement with radiologist assessments (78.35% correct focus; Supplementary Table S2) in the internal validation set. Figure 5 displays four randomly selected representative cases with distinct cardiovascular risk profiles involving: (A) chronic kidney disease, (B) hypertension, (C) hyperlipidemia, and (D) coronary artery disease. As exemplified in Figure 5A, the model's attention heatmap comprehensively covered nearly all AAC calcification areas, yielding a predicted AAC score of 22 points, which closely matched the manual score of 23 points.

### Cardiovascular outcomes

In this 5-year retrospective study, a markedly higher cardiovascular risk was observed in the dialysis cohort, with 17 MACEs (15 patients, including 1 fatality) vs. 7 MACEs (7 patients) in the non-dialysis control cohort. Notably, 93.3% (14/15) of dialysis patients with MACEs and 85.7% (6/7) of non-dialysis cases with MACEs showed clinically significant calcification (AAC score >4). Furthermore, male patients predominated in the cohort (22% female vs. 78% male). Among younger patients with MACEs (age ≤65 years), those on dialysis substantially outnumbered non-dialysis individuals, whereas in the older group (age >65 years), both subgroups were similarly represented. These findings further underscore the prognostic value of vascular calcification assessment (see Table 6 for detailed results).

**Table 6:** MACEs during follow-up according to the need for dialysis.

Parameter	Non-dialysis group (MACE+)	Dialysis group (MACE+)	Statistical values
Demographics			
Age (years)	83 (67–90)	61 (54–79)	*P* = 0.453, *F* = 0.586
Gender (M/F)	5/2	12/3	*P* = 0.673, *F* = 0.183
Calcification			
AAC score (predicted)	8 (3–18)	11 (2–19)	*P* < 0.001, *F* = 10.914
MACE rate			
Entire cohort	7%	15%	E-value = 3.71 (1.42–9.53)
Age ≤65 y (*n* = 9)	0 (0%)	9 (100.0%)	–
Age >65 y (*n* = 13)	7 (46.2%)	6 (53.8%)	–
Male (*n* = 17)	5 (29.4%)	12 (70.6%)	–
Female (*n* = 5)	2 (40%)	3 (60%)	–
MACEs			
Hemorrhagic stroke	1	3	–
Ischemic stroke	2	7	–
Myocardial infarction	4	5	–
Heart failure	0	2	–

## Discussion

This multicenter study compiled and analyzed data for a total of 2,941 cases to develop and test an automated AAC scoring system using deep learning algorithms. The developed scoring system demonstrated both high scoring accuracy and robust clinical reliability in practical application. However, it is important to note that our model was trained and validated on a dataset from a single region, and its generalizability requires further external validation.

### Model evaluation

First, this multicenter study (*n* = 2,941) utilized lumbar/abdominal lateral x-rays per the Kauppila criteria, offering significantly improved data diversity compared with single-center studies (15). Unlike the cohorts in prior studies, which exhibited right-skewed distributions (15, 17, 27), our cohort featured a substantially higher proportion of moderate calcification cases (AAC 5–15), achieving a near-normal sample distribution that enhanced the generalizability of the developed model across all calcification stages.

To address inherent field-of-view (FOV) variations in radiographs caused by technical/anatomical factors, we developed an innovative cascaded framework featuring: (1) a nnU-Net-based lumbar segmentation model, which achieved 92.6% precision in region localization and standardizes inputs by eliminating extra-aortic interference; and (2) a ResNet-based regression architecture (28, 29) that enhances micro-calcification detection (e.g., punctate/arc-shaped patterns) via skip connections while mitigating gradient vanishing. Furthermore, our architectural choices (nnUNet for segmentation and ResNet for regression) were empirically validated through an ablation study, which confirmed their superiority over a strong alternative (VGG16_bn) for this specific task. This systermatic approach to model selection enhances the reliability of our findings.

An important consideration in this study is the treatment of the AAC score as a continuous variable for regression. Although the score is discrete, its foundation in a continuous physical measurement (calcified area) justifies this approach. Our error analyses (Supplementary Figure S3) revealed no substantial bias across the score range, and the majority of prediction errors were minor (±1), indicating that the regression model effectively learned the underlying progression of vascular calcification. However, we acknowleage that the dataset exhibited imbalance, with underrepretation of extreme scores (both very low and very high). This imbalance may partially explain the weak but significant correlation (Spearman's *ρ* = 0.111, *P* < 0.05) between score and absolute error observed in the external validation set.

For clinical reliability, the model achieved clinically acceptable prediction rates (absolute error <4) of 90.0% in the internal validation set and 87.6% in the external validation set. Moreover, the developed model demonstrated excellent agreement with manual scoring in the internal validation set (*R*^2^ = 0.863, ICC = 0.913, weighted Kappa = 0.716), outperforming comparable deep learning models (*R*^2^ = .82) (15), with low prediction errors (MAE = 1.686, MSE = 4.730). External validation confirmed the strong generalizability of our model (*R*^2^ = 0.811, ICC = 0.874, Kappa = 0.644). Subgroup analysis according to AAC severity revealed robust model performance across all categories (average values for accuracy/sensitivity/specificity/NPV/PPV >75%).

The present study also successfully implemented Grad-CAM as a visualization tool for model activation mapping, to achieve dual purposes: (1) enabling clinicians to intuitively verify whether the model accurately identified ROIs and (2) enhancing the transparency and interpretability of AAC score predictions.

### Clinical findings

As a preliminary exploratory study, the primary aim of this study was to provide an initial assessment of the model's performance within a well-characterized yet restricted cohort. This design inherently limits the interpretation of our prognostic findings and their generalizability. Nevertheless, the analysis of 200 systematically sampled AAC-positive cases revealed a significantly higher 5-year MACE incidence in dialysis vs. non-dialysis patients (15.0% vs. 7.0%, *P* < 0.01), with the requirement for dialysis occurring in younger individuals (median age, 61 vs. 83 years). These results not only align with the Kidney Disease Improving Global Outcomes (KDIGO) guidelines (8) and prior evidence (30, 31) but also identify substantial cardiovascular risk (7%) in AAC-positive patients with normal renal function.

Further analysis revealed a significantly higher median AAC score in dialysis-dependent cases that experienced MACEs (11 [IQR 2–19]) vs. non-dialysis cases (8 [IQR 3–18]). Notably, >70% of MACE patients in both groups exhibited moderate-to-severe calcification (AAC score >4), with a particularly high prevalence in the dialysis subgroup (93.3%, 14/15 cases) compared to the non-dialysis subgroup (71.4%, 5/7 cases). These findings corroborate prior research by Gebre et al. and Niu et al. (5, 6, 32), demonstrating that: (1) the AAC score can effectively stratify patients by MACE risk, and (2) patients with moderate-to-severe AAC (AAC score >4) have a substantially elevated cardiovascular risk compared with those with a low AAC score.

### Limitations and future directions

While achieving a significant goal of developing an automated scoring system for AAC, the present study has several limitations. (1) The developed model showed suboptimal performance in cases with severe calcification (AAC score >15) and no/mild calcification (AAC score 0–4), particularly regarding scoring accuracy and subgroup classification. To improve future versions, we plan to incorporate strategies such as cost-sensitive learning and targeted data augmentation, alongside the prospective collection of more cases from under-represented subgroups to enhance model robustness across all AAC severity levels. (2) A potential limitation is the focus on comparing against a single alternative architecture (VGG16_bn). While this comparison robustly justified our choice, future work could expand this to include a broader range of modern achitectures, such as Vision Transformers.

Future directions for this research will include: (1) developing comprehensive calcification quantification metrics with segmental/wall-specific evaluation, complemented by a detailed class-wise analysis to optimize the established AAC scoring system; (2) improving visualization of calcification length/distribution (beyond heatmaps) and validating them via spatial metrics (e.g., Dice coefficient, IoU); (3) incorporating multi-center datasets from diverse regions with variations in imaging protocols, equipment, and demographic backgrounds to enhance model generalizability; and (4) expanding clinical validation through large-scale multicenter studies and multimodal data integration to strengthen cardiovascular risk stratification based on AAC assessment.

## Conclusion

The present study developed a fully automated deep learning-based system for scoring the severity of AAC, and the developed system demonstrated strong agreement with manual ratings in both internal (ICC = 0.913) and external (ICC = 0.874) validation datasets. The developed system enables end-to-end AAC quantification from plain radiographs, offering an efficient solution for population-wide screening.

## Data Availability

The raw data supporting the conclusions of this article will be made available by the authors, without undue reservation.
