# Supplementary material for: Deep learning-based automated quantification system for abdominal aortic calcification: multicenter cohort study for algorithm development and clinical validation
Source: Front Cardiovasc Med. 2025 Oct 21;12:1647882. doi: 10.3389/fcvm.2025.1647882 (PMC12583216; doi:10.3389/fcvm.2025.1647882)
Supplement: Supplementary file 1 [file Supplementaryfile1.docx]

**Materials and methods**

**The VGG16_bn-based AAC scoring regression model**

Similarly, transfer learning was implemented by fine-tuning the ImageNet-pretrained VGG16_bn model weights. Several key modifications were tailored to our task. To handle variable input sizes and stabilize training, an adaptive average pooling layer was used to standardize feature map dimensions to 10×5 prior to classification. The original classifier was replaced with a custom sequential classifier consisting of batch normalization, a dropout layer (p=0.7), and a linear layer outputting the final regression value. This design significantly reduced parameter count compared to the fully-connected layers in standard VGG, mitigating overfitting. To enhance feature representation, we integrated Spatial and Channel Squeeze & Excitation (SCse) attention modules after key convolutional blocks, enabling the model to focus on informative spatial regions and channel features.

The model was optimized using stochastic gradient descent with a momentum of 0.9 and weight decay of 0.0005, with an initial learning rate of 0.01. Training was conducted with a batch size of 32 for 500 epochs using mean squared error (MSE) as the loss function, with early stopping based on validation performance. Validation loss was monitored every 10 epochs, and training was effectively terminated at epoch 330 when validation loss plateaued, indicating no further improvement (Figure S1). We employed an enhanced learning rate scheduling strategy combining linear warmup and cosine annealing, the same as the training of ResNet34-based model.


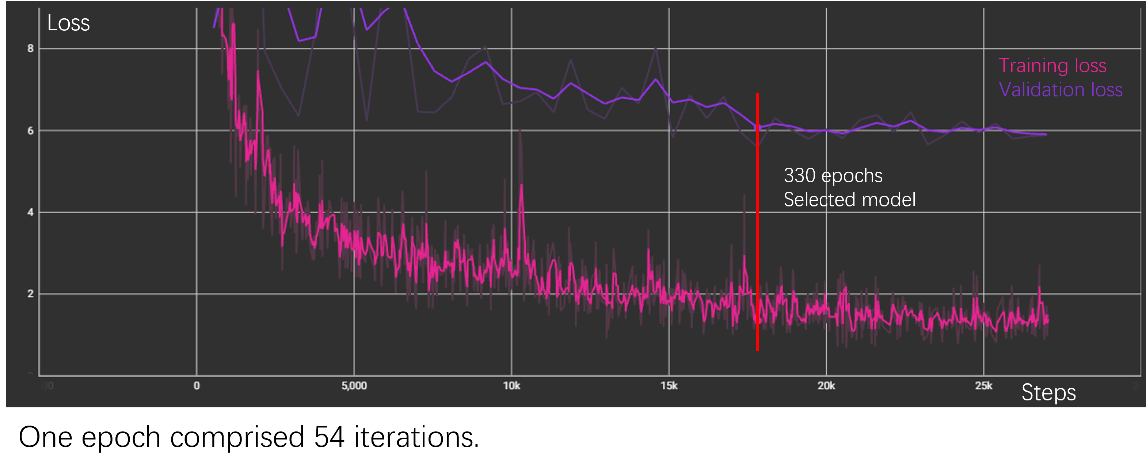


**Figure S1**. Training and validation loss curves of VGG16_bn-based model.


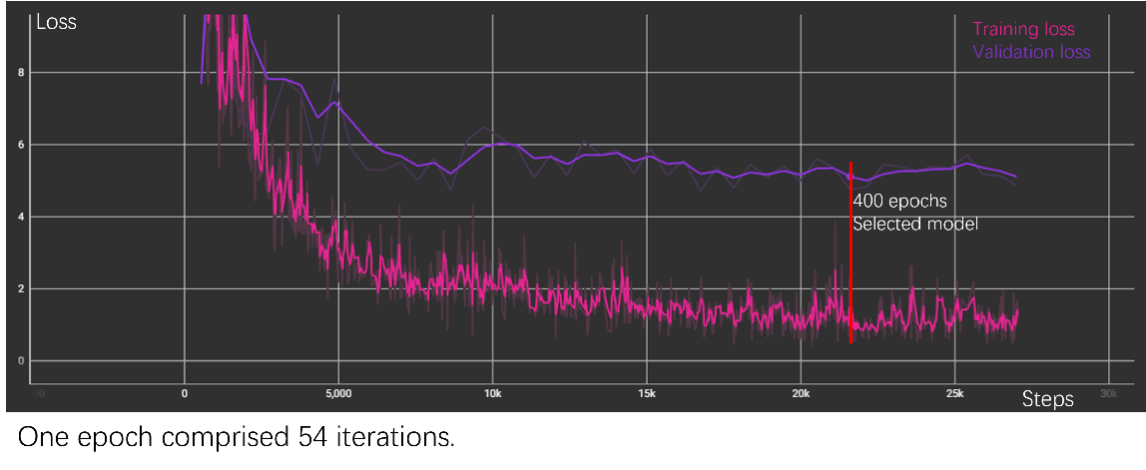


**Figure S2**. Training and validation loss curves of ResNet34-based model.


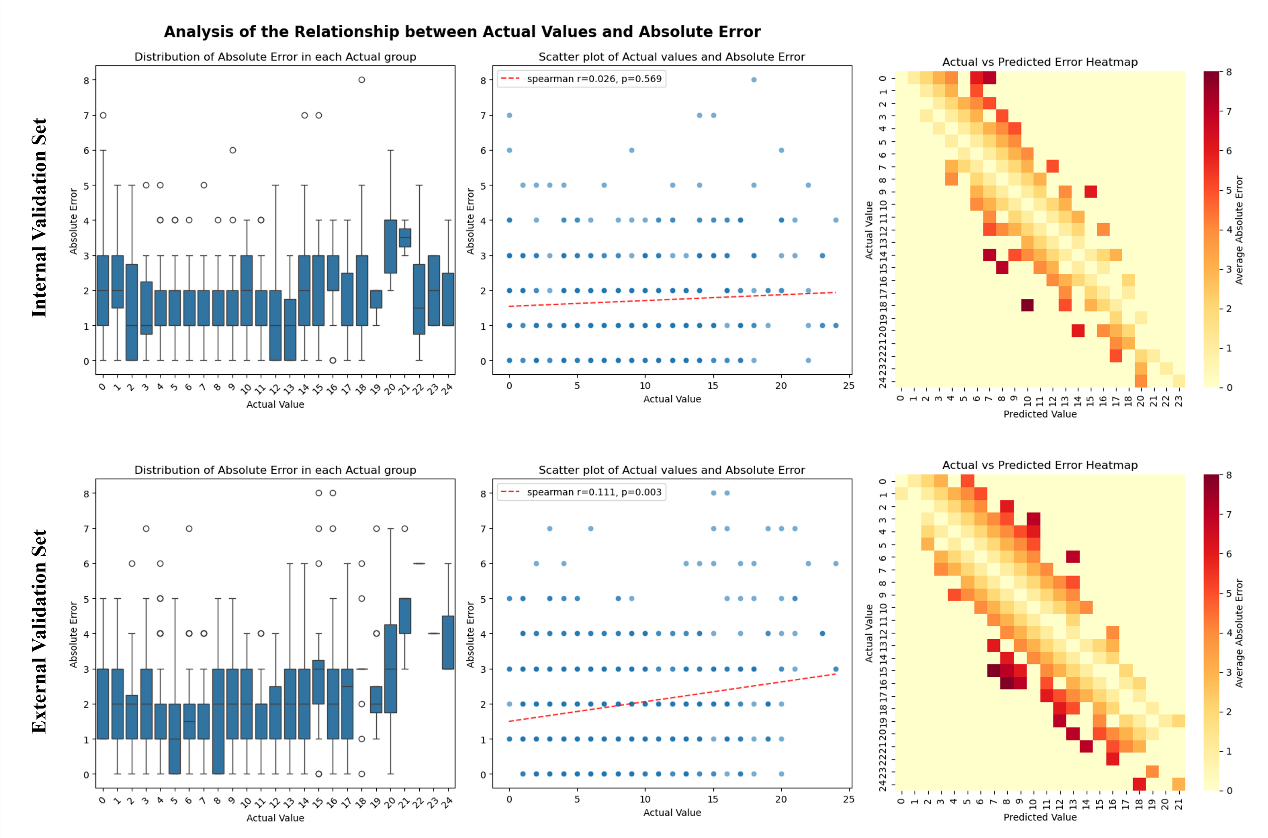


**Figure S3**. Analysis of the Relationship between Actual Values and Absolute Error.

**Table S1**. The comparison results of ResNet34-based and VGG16_bn-based model.

| **Dataset** | **Metrics** | **ResNet34-based model** | **VGG16_bn-based model** |
| --- | --- | --- | --- |
| Internal validation set | MAE | **1.686 (1.571, 1.811)** | 1.875 (1.749，2.006) |
|  | MSE | **4.730 (4.100, 5.393)** | 5.565 (4.881, 6.346) |
|  | Spearman's ρ | **0.923 (0.907, 0.935) *P*<0.001** | 0.920 (0.904, 0.9323)  *P*<0.001 |
|  | R² coefficient | **0.863 (0.835, 0.887) *P*<0.001** | 0.850 (0.823,0.874)  *P*<0.001 |
|  | Clinically Acceptable | **90.0%** | 86.6% |
| External validation set | MAE | **1.920 (1.809, 2.027)** | 2.055 (1.947, 2.164) |
|  | MSE | **5.835 (5.235, 6.486)** | 6.587 (5.924, 7.270) |
|  | Spearman's ρ | **0.888 (0.886, 0.913) *P*<0.001** | 0.880 (0.862,0.895)  *P*<0.001 |
|  | R² coefficient | **0.811 (0.781, 0.835) *P*<0.001** | 0.796 (0.769, 0.823)  *P*<0.001 |
|  | Clinically Acceptable | **87.6%** | 85.8% |

**Table S2.** Qualitative assessment of Grad-CAM visualization accuracy by radiologists.

| **Visual Assessment Category** | **Number of Cases (n)** | **Percentage (%)** | **Interpretation** |
| --- | --- | --- | --- |
| **Correct focus (Aortic wall)** | **369** | **78.35** | Model attention is precisely on the calcified aortic. |
| Partial mismatch | 88 | 18.68 | Model attention includes the calcified aorta but is misaligned or overly diffuse. |
| Major discrepancy | 14 | 2.97 | Model attention is focused on an entirely incorrect anatomical structure. |
| Total | 471 | 100 |  |
